# Benign Lesions in Mucosa Adjacent to Intestinal-Type Sinonasal Adenocarcinoma

**DOI:** 10.4061/2011/230147

**Published:** 2011-04-20

**Authors:** Blanca Vivanco, José Luis Llorente, Jhudit Perez-Escuredo, César Álvarez Marcos, Manuel Florentino Fresno, Mario A. Hermsen

**Affiliations:** ^1^Department of Pathology, Hospital Universitario Central de Asturias, Celestino Villamil s/n, Oviedo, 33006 Asturias, Spain; ^2^Department of Otolaryngology, Instituto Universitario de Oncología del Principado de Asturias (IUOPA), Hospital Universitario Central de Asturias, Edificio H Covadonga 1a Planta Centro, Lab 2, Celestino Villamil s/n, Oviedo,33006 Asturias, Spain

## Abstract

Occupational exposure to wood dust is a strong risk factor for the development of intestinal-type sinonasal adenocarcinoma (ITAC); however, knowledge on possible precursor lesions or biomarkers is limited. Fifty-one samples of tumor-adjacent mucosa and 19 control samples of mucosa from the unaffected fossa of ITAC patients were evaluated for histological changes and p53 protein expression. Mild dysplasia was observed in 14%, cuboidal metaplasia in 57%, intestinal metaplasia in 8%, squamous metaplasia in 24%, and cylindrocellular hyperplasia in 53% of cases. P53 immunopositivity was generally weak occurring most frequently in squamous metaplasia. Wood dust etiology did not appear of influence on the histological changes, but p53 showed a tendency for higher positivity. Dysplasia adjacent to tumor was indicative of subsequent development of recurrence. In conclusion, precursor lesions do occur in mucosa adjacent to ITAC. This is clinically important, because it may justify the screening of high-risk individuals such as woodworkers.

## 1. Introduction

Intestinal-type sinonasal adenocarcinomas (ITACs) are epithelial tumors of the nasal cavities and paranasal sinuses, often related to professional exposure to wood dust. It is a rare tumor representing 8–25% of all malignant sinonasal tumors [1, 2]. According to the WHO histological classification [3], two main categories are recognized: intestinal-type and non-intestinal-type adenocarcinoma. The latter is not related to professional wood dust exposure and is not the subject of this paper. Based on classifications of Barnes and Kleinsasser [2, 4], five pathological types of sinonasal ITAC are distinguished: papillary or papillary tubular cylinder cell I (PTCC-I), colonic (PTCC-II), solid (PTCC-III), mucinous (alveolar goblet and signet ring), and mixed (transitional). The most frequent type is colonic (40%), followed by solid (20%), papillary (18%), and mucinous and mixed type (together 22%) [3]. 

In the northern part of Spain, the incidence is 0.19 cases/100.000 inhabitants per year [5]. It is located most frequently (85%) in the ethmoid sinus and the upper part of the nasal cavity [6, 7]. Distant or lymph node metastases are exceptional, while local recurrences constitute the main cause of death among patients [8, 9]. The median age of onset lies between 50 and 60 years [4, 10] and in wood dust-related tumors even earlier [10]. Men develop ITAC four times more frequently than women, reflecting the occupational hazard implicated [4]. 

In the clinic, ITAC often appear as indolent, slow-growing tumors with unspecific unilateral symptoms (occasionally bilateral) normal to this site of origin, such as nasal obstruction, epistaxis, or rhinorrhea [9]. Frequently they are confused with chronic inflammation (rhinitis, sinusitis) or benign tumors. Because of this, diagnosis is often late, with an interval of 6–8 months from the first symptoms to diagnosis. By then the tumor can already be advanced stage with orbital, intracranial, oral, or facial soft tissue extension. Therefore, there is a need for better ways of prevention and early diagnosis.

Previous reports have focussed on finding precursor lesions in series of sinonasal mucosa samples from persons at high risk of developing ITAC, that is, woodworkers. Aiming to find stronger indications, in this study we analyzed normal sinonasal mucosa of patients who have already developed ITAC. In addition to the histological evaluation, we studied p53 protein expression as a possible marker for early neoplastic transformation.

## 2. Material and Methods

### 2.1. Patients and Samples

Fifty-one paraffin tissue samples were taken from mucosa adjacent to the tumor, with presence of both tumor and normal tissues in the same tissue block. Nineteen control samples were obtained from the healthy, unaffected fossa of patients with ITAC. In 9 cases we obtained a sample from both adjacent tissue and the other fossa. All samples were collected from previously untreated patients seen between 1990 and 2009. Informed consent was obtained from all patients, and the study was approved by the ethical committee of our institute. 

Of the 51 cases, 1 patient was female and 50 male, with a mean age of 65 years (45–92). Forty-five patients had occupational exposure to wood dust with a median of 35 years (range: 4–55 years), and 27 were tobacco and alcohol users. Fifteen tumors were stage I, five stage II, seventeen stage III, eight stage IVa, and six stage IVb. No patient had metastases at the time of diagnosis. According to the WHO histological classification [3], our series comprised of 7 papillary type or PTCC-I (papillary tubular cylinder cell I), 23 colonic (PTCC-II), 4 solid (PTCC-III), and 17 mucinous type tumors. All patients underwent radical surgery, and in all cases resection margins were free of tumor. Forty of the patients received complementary radiotherapy. Follow-up information was available with a median of 30 months (range: 1–242). The 5-year survival rate was 53%. Twenty-seven patients developed local recurrence, and five other had metastases in the brain. At the time of writing, 26 patients were alive, 21 died of disease, and 4 died of other causes.

Of the 19 controls, 2 patients were female and 17 male, with a mean age of 69 years (45–78). Seventeen had occupational exposure to wood dust with a median of 35 years (range: 1–50 years), and 9 were tobacco and alcohol users.

### 2.2. Histological Examination

H&E stained paraffin sections were grouped as: (1) mucosa adjacent to tumor and (2) control mucosa from the other unaffected fossa of a patients with ITAC. The surface epithelium of all samples was evaluated for the presence of respiratory epithelium, dysplasia, cuboid metaplasia, squamous metaplasia, and cylindrocellular hyperplasia (including basal cell hyperplasia, mucrosecretory hyperplasia, and transitional type hyperplasia). Intestinal metaplasia was evaluated with the help of cytokeratin 20 immunostaining. The seromucinous glands were analyzed for signs of dysplasia and mucosecretory hyperplasia.

### 2.3. P53 Immunohistochemistry

Immunohistochemistry was performed on 4 *μ*m paraffin embedded sections with the antibodies anti-p53 clone DO-7 and cytokeratin 20 clone K.208 (DAKO, Glostrup, Denmark) using an automatic staining workstation (Dako Autostainer, Dako Cytomation, Glostrup, Denmark) with the Envision system and diaminobenzidine chromogen as substrate. P53 expression was evaluated both in surface epithelium and in seromucinous glands and scored in 4 categories: 0–10%, 10–25%, 25–50%, and 50–100%.

### 2.4. Statistical Analysis

Possible correlations were statistically analyzed by SPSS 12.0 software for Windows (SPSS Inc. Illinois, USA), using the Fisher Exact and Pearson Chi2 test. Kaplan-Meier analysis was performed for estimation of survival, comparing distributions of survival through the logarithmic range test (log-rank test). *P*  values below  .05 were considered significant.

## 3. Results and Discussion

### 3.1. Histological Changes

In 6 of 51 (12%) samples, no histological abnormality was observed in the mucosa adjacent to ITAC. Of these 6 cases, 3 ITACs were stage T1, 1 T2, 1 T3, and 1 T4b and concerned 3 colonic and 3 mucinous type tumors. Over half of the cases (24/45) showed more than one histological abnormality. 

**Figure 1:**
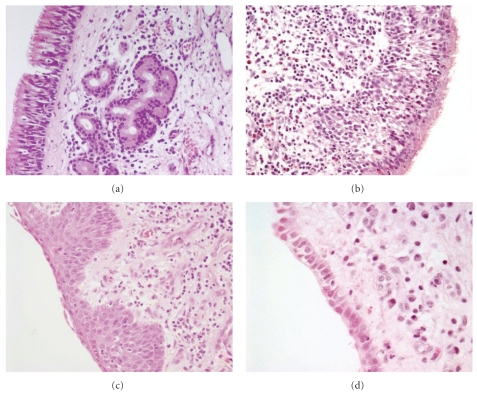
Microphotographs of the different histological changes observed adjacent to the tumor. (a) Normal respiratory mucosa; (b) basal cell hyperplasia; (c) squamous metaplasia; (d) cuboid metaplasia. H&E staining, original magnification 200x (a, b, and c) and 400x (d).

**Figure 2:**
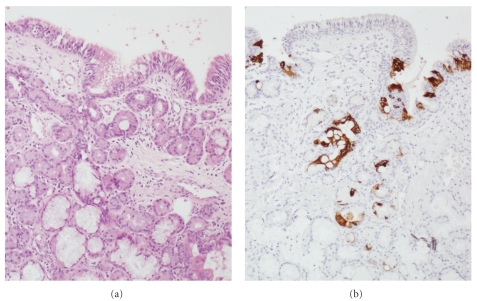
Microphotographs of intestinal metaplasia, both in the surface epithelium and in the seromucous glands. (a) H&E staining. (b) Immunohistochemical staining of CK20, original magnification 200x (a and b).

**Figure 3:**
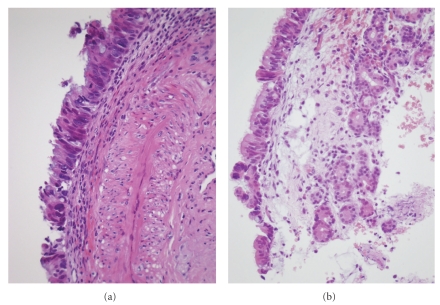
Microphotographs of two cases with dysplasia. (a) Mild dysplasia in the surface epithelium. (b) Mild dysplasia both in the surface epithelium and in the seromucous glands. H&E staining, original magnification 400x (a and b).

In the surface epithelium of the remaining 45 samples, we observed mild dysplasia in 7 (16%), cuboid metaplasia in 29 (64%), squamous metaplasia in 12 (27%), and cylindrocellular hyperplasia in 27 (60%) cases (Figure 1). CK20 immunostaining, indicative for intestinal metaplasia (Figure 2), resulted in 4 (9%) positive cases. In the seromucous glands, we detected 10 (20%) cases with mild dysplasia (Figure 3) and 17 (33%) cases with hyperplasia. Dysplasia in the respiratory mucosa correlated with dysplasia in the seromucous glands (Fisher exact chi2: *P* = .029). The 4 cases with CK20 immunopositivity in the surface mucosa also showed positivity in the seromucous glands (Figure 2). Two published studies that evaluated mucosa adjacent to ITAC differ very much, and our data seem to take position in between these two studies. For instance, Valente et al. [11] found no dysplasia and no squamous metaplasia in 15 samples, whereas Wilhelmsson and Lundh [12] reported 73% and 23%, respectively, in 22 samples. Intestinal metaplasia in mucosa adjacent to ITAC, detected in 4/51 of our cases by aid of CK20 immunostaining, has been reported previously in 1/10 [17] and in 4/12 [18] cases.

In normal mucosa of the unaffected fossa of ITAC patients, we found 9 of 19 (47%) cases without abnormalities and in general much lower frequencies of histological changes than in the mucosa adjacent to tumor (Table 1). This is similar to the literature concerning mucosa samples obtained from woodworkers who had not developed ITAC, with the exception of squamous metaplasia, which in our series was less frequent [11, 13–16] (Table 1). No CK20 positivity or intestinal metaplasia was observed in these 19 control samples, which is in agreement with Palomba et al. who reported complete absence of CK20 in a series of 139 normal mucosa samples from leather workers [15].

Although the number of cases without wood dust etiology was very low, our data suggest that wood dust exposure does not cause specific histological changes, except perhaps for cylindrocellular hyperplasia. This is confirmed by Wolf et al. who studied a large series of samples [13]. On the other hand, some studies reported a higher frequency of squamous metaplasia in woodworkers compared to controls [14–16]. Tobacco smoking did not associate with any of the histological changes.  Table 3 shows that the histological changes of the mucosa adjacent to tumor were not related to the histological tumor type or T stage. Only mucosa adjacent to papillary type ITAC seemed to harbour less cuboid metaplasia and more hyperplasia compared to the other three tumor types. Dysplasia was present in 5 of 27 (19%) patients that developed recurrence and only in 2 of 24 (8%) that did not; however, due to the low number of cases, this did not reach statistical significance (Fisher exact chi2 *P* = .261). In addition, all 4 patients with CK20 immunopositivity (i.e., intestinal metaplasia) in the mucosa adjacent to tumor had a tumor recurrence. Neither of the histological aberrations were related to overall or disease-free survival. To our knowledge, the relation between tumor and clinical characteristics and histological changes in mucosa adjacent to the tumor has not been studied previously in the literature.

### 3.2. P53 Expression

**Figure 4:**
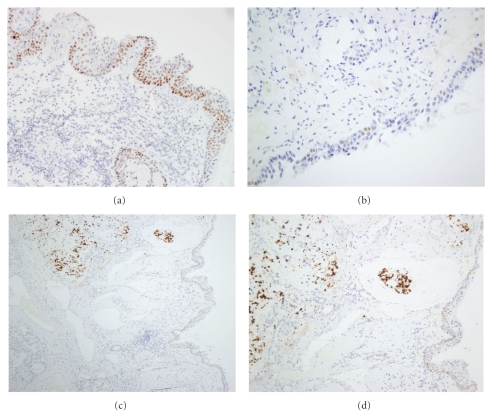
Immunohistochemical analysis of p53 expression. (a) Moderate, diffuse staining in basal and suprabasal cells in squamous metaplasia; (b) weak, focal staining in mild dysplasia; (c) strong staining in a mucinous type ITAC, with weak/moderate staining in adjacent immature metaplasia and seromucous glands; (d) details of (c). H&E staining, original magnification 200x (a, b, and d) and 100x (c).

In general, p53 positivity was weak and occurred in a low percentage of cells (Figure 4), in the mucosa adjacent to ITAC and especially in the mucosa from the other unaffected fossa. In normal respiratory epithelium p53 expression was absent. Among the distinct histological lesions, squamous metaplasia showed the strongest positivity; interestingly dysplasia and cuboid metaplasia demonstrated a very low expression. P53 positivity in the surface epithelium was always accompanied by p53 positivity in the seromucous glands, but there was no correlation with p53 expression in the adjacent tumor (Pearson chi2 *P* = .923). It may be speculated that the relatively low level of p53 positivity in the mucosa adjacent to tumor does not indicate TP53 gene mutation, but rather an upregulation of functional p53 in response to inflammatory signals in the wood dust-exposed sinonasal epithelium.

Our data, summarized in Table 2, are similar to a previous study studying ITAC, adjacent mucosa, and wood-exposed controls [11]. In addition, we confirmed a tendency of higher p53 positivity in the samples from patients with wood etiology, but not in the number of samples with some p53 positivity. Studying both ITAC and sinonasal squamous carcinoma, Holmila and coworkers also noted a trend for higher p53 expression in wood dust-related patients [19]. We found no difference in p53 expression between smokers and nonsmokers, and this was also seen by Holmila et al. [19].

When analyzing the p53 results in relation to the adjacent histological tumor type, we noted a higher expression in mucosa adjacent mucinous type ITAC (Table 3). However, p53 expression in the 51 ITAC tumors was different among the four histological subtypes. Finally, p53 positivity in mucosa adjacent to tumor did not correlate to the tumor T stage nor to the development of recurrence during follow-up.

## 4. Conclusion

ITAC represents an important occupational health problem with serious consequences, needing better ways of prevention, early diagnosis, and treatment. Despite a clear etiology, it is still unknown how they develop. The present model suggests that inhaled wood dust particles larger than 5 *μ*m become trapped in the mucosa of the middle turbinate and ethmoid [6, 7] and weaken the ciliar function of the nasal cells which prolong their contact with the mucosa and so their possible carcinogenic effects [7]. Since wood dust does not have direct mutagenic properties, it may be hypothesized that prolonged exposure to and irritation by wood dust particles stimulate cellular turn-over by inflammatory pathways. Chronic inflammation is recognized as an important mechanism in tumor initiation and progression in various cancer types, such as colorectal (inflammatory bowel disease), stomach (gastritis), and esophageal carcinoma (Barrett esophagus). Recently, Holmila et al. [19] showed that wood dust-related nasal adenocarcinomas have elevated COX2 levels, indicating a role for chronic inflammation in the tumorigenesis of ITAC. In addition, it has been shown that the TP53 mutation profile found in ITAC fits a causal role for reactive oxygen species, such as generated in chronic inflammatory processes [20]. It will be interesting to further investigate the possible role of chronic inflammation in sinonasal mucosa of woodworkers.

Epithelial cancers are frequently preceded and accompanied by precursor changes in the tissue histology, of which dysplasia is generally believed to be a true premalignant lesion. In previous studies on normal mucosa of woodworkers, dysplasia was found in around 10% of cases [13–16]. In our study concerning mucosa adjacent to tumor, we had expected to find a higher percentage, but we found a similar number of dysplasias. It may be speculated that dysplasia indeed does precede ITAC in a majority of cases but that it becomes destroyed by the growth of the ITAC tumor mass. However, when we contrasted the presence of dysplasia of stage T1 tumors to larger, later stage tumors, we saw no differences (Table 3). Wilhelmsson et al. observed cuboid metaplasia often together with dysplasia and suggested this to be a precursor to ITAC [12, 21]. We found a high percentage of cuboid metaplasia. In addition, of 5 cases with dysplasia accompanied by cuboid metaplasia, 4 developed a recurrence. Our data may therefore be in accordance with this suggestion. Alternatively, it has been proposed that ITAC, which is predominantly CK20 positive, evolves from intestinal metaplasia [18]. In our series 4 cases had CK20-positive mucosa adjacent to ITAC and all 4 developed a recurrence and may also support this theory. These two notions are not necessarily contradictory. Kennedy et al. hypothesized that normal respiratory mucosa first undergoes cuboid metaplasia, followed by intestinal metaplasia, and then possibly through dysplasia develops into ITAC [17].

Whatever the tumor-initiating causes, cancer arises through a multistep sequence in which histological changes are accompanied by the accumulation of genetic aberrations. The analysis of genetic or epigenetic aberrations in these precursor lesions will give a more definitive insight in their possible role in ITAC development.

## Figures and Tables

**Table 1:** Histological changes in mucosa adjacent to ITAC and in controls and comparison to the literature.

		This study				[11]		[12]	[13]		[14]		[15]		[16]	
	Tissue sample	Adjacent		Control		Adjacent		Adjacent	Mucosa		Mucosa		Mucosa		Mucosa	
	Wood exposure	Yes	No	Yes	No	Yes	No	Yes	Yes	No	Yes	No	Yes*	No	Yes	No
	Number of samples	45	6	17	2	10	5	22	144	31	65	68	139	16	113	54
Surface epithelium	Dysplasia	6 (13%)	1 (17%)	1 (6%)	0	0 (0%)	0 (0%)	16 (73%)	7 (5%)	1 (3%)	0 (0%)	0 (0%)	37 (27%)	0	14 (12%)	1 (2%)
	Cuboidal metaplasia	25 (56%)	4 (67%)	4 (24%)	1 (50%)			19 (86%)	40 (28%)	5 (16%)						
	Squamous metaplasia	10 (22%)	2 (33%)	1 (6%)	0	0 (0%)	0 (0%)	5 (23%)	57 (40%)	16 (52%)	40 (61%)	5 (7%)	90 (65%)	2 (13%)	41 (40%)	17%
	Hyperplasia	26 (58%)	1 (17%)	4 (24%)	1 (50%)				59 (41%)	6 (19%)			30 (22%)	1 (6%)		
	Intestinal metaplasia	3 (7%)	1 (17%)	0	0											

Seromucinous glands	Dysplasia	8 (18%)	2 (33%)	0	0											
	Hyperplasia	14 (31%)	3 (50%)	7 (41%)	0											

*Exposure to leather dust.

**Table 2:** P53 immunopositivity in mucosa adjacent to ITAC and controls and comparison to the literature.

	This study						[11]					
	Tumor	Tumor	Adjacent	Adjacent	Control	Control	Tumor	Tumor	Adjacent	Adjacent	Control	Control
Wood exposure	Yes	No	Yes	No	Yes	No	Yes	No	Yes	No	Yes	No
Number of samples	45	6	45	6	17	2	10	5	10	5	68	81
Overall	68% (*n* = 33)	27% (*n* = 4)	24% (*n* = 22)	27% (*n* = 2)	35% (*n* = 1)	0%	69%	15–28%	12%	1%	12%	2%
Dysplasia			17% (*n* = 1)	0%	0%	0%						
Metaplasia cuboidal			17% (*n* = 1)	0%	35% (*n* = 1)	0%						
Metaplasia squamous			37% (*n* = 6)	37% (*n* = 1)	0%	0%					28%	8%
Hyperplasia			27% (*n* = 2)	0%	0%	0%						
Respiratory epithelium			21% (*n* = 12)	17% (*n* = 1)	0%	0%						
Seromucinous glands			24% (*n* = 22)	27% (*n* = 2)	NA	NA					12%	1%

P53 immunopositivity is presented as the mean % of positive cells calculated from the cases that showed some positivity (the number of cases is given between brackets). NA: not applicable for lack of representation.

**Table 3:** Histological changes and p53 expression in relation to adjacent tumor type and tumor T stage.

	Nr	Dysplasia	Cuboid	Squamous	Hyperplasia	p53 Adjacent mucosa				p53 tumor	
			Metaplasia	Metaplasia		0–10%	10–25%	25–50%	50–100%	<10%	>10%
Papillary	7	1 (17%)	2 (29%)	1 (17%)	5 (83%)	2 (29%)	4 (57%)	1 (14%)	0	1 (14%)	6 (84%)
Colonic	23	5 (22%)	16 (70%)	5 (22%)	5 (22%)	16 (70%)	6 (26%)	1 (4%)	0	3 (13%)	20 (87%)
Solid	4	0	3 (75%)	0	0	2 (50%)	2 (50%)	0	0	0	4 (100%)
Mucinous	17	1 (6%)	8 (47%)	6 (35%)	6 (35%)	7 (41%)	4 (24%)	5 (29%)	1 (6%)	7 (41%)	10 (59%)

T1	15	2 (13%)	9 (60%)	2 (13%)	7 (47%)	11 (73%)	3 (20%)	1 (7%)	0	4 (27%)	11 (73%)
T2	5	1 (20%)	3 (60%)	0	2 (40%)	2 (40%)	2 (40%)	1 (20%)	0	2 (40%)	3 (60%)
T3	17	2 (12%)	9 (53%)	6 (35%)	11 (65%)	10 (59%)	5 (29%)	1 (6%)	1 (6%)	4 (24%)	13 (76%)
T4	14	2 (14%)	8 (57%)	4 (29%)	7 (50%)	4 (29%)	7 (50%)	3 (21%)	0	2 (14%)	12 (86%)

P53 immunopositivity is presented as the number of cases that showed some positivity.
